# Supplementary material for: Crisis leadership and strategic decisions in Swedish maternity care during the COVID-19 pandemic: A deductive analysis from the COPE staff project
Source: PLoS One. 2026 May 22;21(5):e0346625. doi: 10.1371/journal.pone.0346625 (PMC13196918; doi:10.1371/journal.pone.0346625)
Supplement: S1 Table — (DOCX) [file pone.0346625.s001.docx]

## Table 1 Informants

| Number | Title | Size of the operation ^[[1]](#footnote-1)^ | Own profession |
| --- | --- | --- | --- |
| 1 | Operations manager | Larger unit | Physician |
| 2 | Care unit manager | Larger unit | Midwife |
| 3 | Operations manager | Medium-sized unit | Physician |
| 4 | Care unit manager | Larger unit | Midwife |
| 5 | Operations manager | Larger unit | Physician |
| 6 | Operations manager | Larger unit | Physician |
| 7 | Operations manager | Larger unit | Physician |
| 8 | Operations manager | Medium-sized unit | Physician |
| 9 | Care unit manager | Smaller unit | Midwife |
| 10 | Operations manager | Medium-sized unit | Physician |
| 11 | Operations manager | Medium-sized unit | Physician |
| 12 | Care unit manager | Medium-sized unit | Midwife |
| 13 | Care unit manager | Medium-sized unit | Midwife |
| 14 | Care unit manager | Medium-sized unit | Midwife |
| 15 | Medical director | Medium-sized unit | Physician |
| 16 | Medical director | Medium-sized unit | Physician |
| 17 | Care unit manager | Medium-sized unit | Midwife |
| 18 | Medical director | Smaller unit | Physician |

1. Larger unit above 3000 births per year

   Medium-sized unit 1500-3000 births per year

   Smaller unit less than 1500 births per year [↑](#footnote-ref-1)
