# Supplementary material for: Crisis leadership and strategic decisions in Swedish maternity care during the COVID-19 pandemic: A deductive analysis from the COPE staff project
Source: PLoS One. 2026 May 22;21(5):e0346625. doi: 10.1371/journal.pone.0346625 (PMC13196918; doi:10.1371/journal.pone.0346625)
Supplement: S2 Table — (DOCX) [file pone.0346625.s002.docx]

| **Domain** | **Construct** |
| --- | --- |
| I. Innovation domain | A. Innovation source |
|  | B. Innovation Evidence Base |
|  | C. Innovation Relative Advantage |
|  | D. Innovation Adaptability |
|  | E. Innovation Trialability |
|  | F. Innovation Complexity |
|  | H. Innovation Costs |
| II. Outer Setting domain | A. Critical Incidents |
|  | B. Local Attitudes |
|  | C. Local Conditions |
|  | D. Partnerships & Connections |
|  | E. Policies & Laws |
|  | F. Financing |
|  | G. External Pressure |
| III. Inner Setting domain | A. Structural Characteristics |
|  | B. Relational Connections |
|  | C. Communications |
|  | D. Culture |
|  | E. Tension for Change |
|  | J. Available Resources |
|  | K. Access to Knowledge & Information |
| IV. Individuals domain | A. Need |
|  | B. Capability |
|  | C. Opportunity |
|  | D. Motivation |
| V. Implementation Process domain | A. Teaming |
|  | B. Assessing Needs |
|  | C. Assessing Context |
|  | D. Planning |
|  | E. Tailoring Strategies |
|  | F. Engaging |
|  | H. Reflecting & Evaluating |
|  | I. Adapting |

# **Table 2 Domains and Constructs**

Domains and constructs according to the CFIR(1) illustrate the five domains related to how managerial

decisions were made and implemented in maternity care units in Sweden during the Covid-19 pandemic. 1. Damschroder LJ, Reardon CM, Widerquist MAO, Lowery J. The updated Consolidated Framework for Implementation Research based on user feedback. Implement Sci IS. 29 oktober 2022;17(1):75.
