# Supplementary material for: Crisis leadership and strategic decisions in Swedish maternity care during the COVID-19 pandemic: A deductive analysis from the COPE staff project
Source: PLoS One. 2026 May 22;21(5):e0346625. doi: 10.1371/journal.pone.0346625 (PMC13196918; doi:10.1371/journal.pone.0346625)
Supplement: S3 Table — (DOCX) [file pone.0346625.s003.docx]

## Table 3. Example of the Analytical Process

| **Meaning unit** | **Code** | **Construct** | **Domain** |
| --- | --- | --- | --- |
| *...my colleagues and I, fellow heads of departments, felt a lot of pressure from the politicians to loosen these visitation restrictions because they, in turn, surely faced a lot of pressure from the public... Even though they said they wouldn't interfere with the medical aspect, they did.* | Political pressure to lift visitation restrictions | G: External pressure | Outer setting |
| *We have summarized the pandemic a few times at the clinic, noting both strengths and concerns. One of the strengths is that there is even less silo thinking now. It's very clear that there is more collaboration. Additionally, transparency has been necessary to prioritize health care effectively.* | Pandemic led to increased collaboration and transparency | H: Reflecting & Evaluating | Implementations  process |
