# Supplementary material for: Crisis leadership and strategic decisions in Swedish maternity care during the COVID-19 pandemic: A deductive analysis from the COPE staff project
Source: PLoS One. 2026 May 22;21(5):e0346625. doi: 10.1371/journal.pone.0346625 (PMC13196918; doi:10.1371/journal.pone.0346625)
Supplement: S1 File — (DOCX) [file pone.0346625.s004.docx]

# **Interview Guide Based on CFIR**

## **Introductory Questions**

- Can you start by telling me a bit more about your leadership role?
- How many years have you worked at this clinic in total?
- How many years have you been a manager at the clinic?
- How long have you been in your current position?
- How many subordinates do you have?
- What assignments have you had during the past year (during the pandemic)?
- Can you describe a decision about a change during the pandemic that stands out to you? It could be a decision that was particularly important for the organization, especially difficult to make, or one that had significant consequences.

## **Intervention Characteristics**

### **Intervention Source**

- Who was involved in making the decision about the change?

### **Evidence Strength & Quality**

- What kind of evidence or knowledge base was used to support the decision?

### **Relative Advantage**

- What differences in decision-making processes were there during the COVID-19 pandemic compared to normal circumstances?
- Are there advantages/disadvantages to the changes in the decision-making process?

### **Adaptability**

- What changes were required for the decisions to work in practice?
- Were they feasible to implement?

### **Design Quality & Packaging**

- What is your opinion on how the decisions were communicated to staff?
- To managers and the leadership team?
- What channels did you use to disseminate the information?
- Were they sufficient?

### **Cost**

- Were budget and costs considered when making the decisions?

## **Outer Setting**

### **Patient Needs & Resources**

- Have you received feedback on the decisions that were made?
- What do the staff think?
- What does the leadership team think?
- What do patients and their relatives think?

## **Inner Setting**

### **Structural Characteristics**

- What structural changes were required to implement the decisions?
- Changes in clinical operations? (Staff composition, staff in risk groups, layout of the premises?)
- Policy changes?
- Changes in information transfer or record-keeping?
- Other changes?

### **Networks & Meetings**

- When you need support in making a decision, who do you usually turn to?
- Can you give an example based on the decisions made in relation to the pandemic?

### **Implementation Culture**

- How receptive do you think the organization has been to implementing new decisions related to the pandemic?

### **Compatibility**

- How have the decisions affected work processes, improvement efforts, and ongoing studies?

### **Relative Priority**

- How do you balance prioritizing and making trade-offs regarding which activities can continue and which need to be paused or stopped based on the decisions you’ve made?

### **Readiness for Implementation**

### **Leadership Engagement**

- What kind of information exchange occurred at the national level at the start of the pandemic?
- Were changes and implementation discussed among department heads in general?
- Within VGR (Region Västra Götaland)?
- Within professional networks?
- Has there been region-wide leadership support for implementing measures related to the pandemic?
- Can you give examples of the support you received? Decision-making support? Financial support?
- What additional support would you need?
- How have you documented the decisions and measures that were implemented?
- Are these accessible to staff? To the public?

## **Characteristics of Individuals**

### **Knowledge & Beliefs about the Intervention**

- Now that some time has passed, how do you view the decisions that were made?
- Have they been effective?

## **Process**

### **Champions**

- Besides the leadership team, are there individuals in the organization who contributed significantly to successful implementation?

### **Reflecting & Evaluating**

- Do you receive regular reports on how the organization is functioning?
- How is the evaluation carried out? By whom?
