# Supplementary material for: Crisis leadership and strategic decisions in Swedish maternity care during the COVID-19 pandemic: A deductive analysis from the COPE staff project
Source: PLoS One. 2026 May 22;21(5):e0346625. doi: 10.1371/journal.pone.0346625 (PMC13196918; doi:10.1371/journal.pone.0346625)
Supplement: Attachment — Submitted filename: Response to reviewers _rebuttal letter_leading in crisis.docx [file pone.0346625.s006.docx]

**Response to the editor and reviewers**

*We appreciate the opportunity to revise the manuscript with the updated title. We would like to extend our sincere thanks to the editor and reviewers for their time and for the valuable and constructive feedback, which we believe has helped improve the quality of the work. Our responses to the comments are presented below.*

**Comments from the Editor**

Journal Requirements:

1. Please ensure that your manuscript meets PLOS ONE's style requirements, including those for file naming. The PLOS ONE style templates can be found at

<https://journals.plos.org/plosone/s/file?id=wjVg/PLOSOne_formatting_sample_main_body.pdf> and

[https://journals.plos.org/plosone/s/file?id=ba62/PLOSOne_formatting_sample_title_authors_affiliations.pdf](https://track.editorialmanager.com/CL0/https:%2F%2Fjournals.plos.org%2Fplosone%2Fs%2Ffile%3Fid=ba62%2FPLOSOne_formatting_sample_title_authors_affiliations.pdf/1/010f019806765347-60962b0b-5aea-4311-8f7c-71c0c6cb4f00-000000/bS1r1FiqCu-0JMfNifmRcf2iwWN7LJsZlnJ6tAoBn7Q=219)

*Answer: Thank you. We have ensured that the manuscript meets PLOS ONE's style requirements.*

2. Thank you for stating the following financial disclosure:
“FORMAS – the Swedish Research Council for Sustainability (2020-02767) – for funding the research”
Please include this amended Role of Funder statement in your cover letter; we will change the online submission form on your behalf.

*Answer: The funders had no role in study design, data collection and analysis, decision to publish, or preparation of the manuscript. We have included the amended Role of Funders in our cover letter.*

3. We note that you have indicated that there are restrictions to data sharing for this study. For studies involving human research participant data or other sensitive data, we encourage authors to share de-identified or anonymized data. However, when data cannot be publicly shared for ethical reasons, we allow authors to make their data sets available upon request. For information on unacceptable data access restrictions, please see <http://journals.plos.org/plosone/s/data-availability#loc-unacceptable-data-access-restrictions>.

Before we proceed with your manuscript, please address the following prompts:

a) If there are ethical or legal restrictions on sharing a de-identified data set, please explain them in detail (e.g., data contain potentially identifying or sensitive patient information, data are owned by a third-party organization, etc.) and who has imposed them (e.g., a Research Ethics Committee or Institutional Review Board, etc.). Please also provide contact information for a data access committee, ethics committee, or other institutional body to which data requests may be sent.

*Answer: Thank you for raising this important point. We fully support open sharing of de-identified data. However, due to the extremely limited recruitment pool, large parts of the dataset includes potentially identifiable information. Nevertheless, we are able to share relevant excerpts from the transcripts upon request, pending approval from the University of Gothenburg, contact* [*Karin.ahlberg@gu.se*](mailto:Karin.ahlberg@gu.se)

Please update your Data Availability statement in the submission form accordingly.

*Answer: We have updated the Data Availability statement in the submission form accordingly.*

4. In the online submission form, you indicated that [Data contains potentially identifying and sensitive information, and there are ethical restrictions to sharing the data file as it includes individual interviews with participants. For data request, please contact The Swedish Ethical Review Authority: [registrator@etikprovning.se](mailto:registrator@etikprovning.se)].
All PLOS journals now require all data underlying the findings described in their manuscript to be freely available to other researchers, either 1. In a public repository, 2. Within the manuscript itself, or 3. Uploaded as supplementary information.
This policy applies to all data except where public deposition would breach compliance with the protocol approved by your research ethics board. If your data cannot be made publicly available for ethical or legal reasons (e.g., public availability would compromise patient privacy), please explain your reasons on resubmission and your exemption request will be escalated for approval.

*Answer: Thank you for highlighting this important requirement. We fully support open science principles and the sharing of de-identified data. However, due to the highly limited recruitment pool and the sensitive nature of the interview material, the dataset contains potentially identifiable information that cannot be fully anonymized without compromising participant confidentiality. As such, public deposition would not be in compliance with the ethical approval granted for this study.*

*Nevertheless, we are committed to transparency and will make relevant excerpts from the transcripts available* *upon reasonable request, pending approval from the University of Gothenburg. Researchers interested in accessing such material may contact Professor Karin Ahlberg (Head of Department), who serves as the institutional representative for data access matters, at karin.ahlberg@gu.se.*

*We have updated our data availability statement accordingly and respectfully request an exemption from public deposition in line with PLOS data sharing policy.*

5. When completing the data availability statement of the submission form, you indicated that you will make your data available on acceptance. We strongly recommend all authors decide on a data sharing plan before acceptance, as the process can be lengthy and hold up publication timelines. Please note that, though access restrictions are acceptable now, your entire data will need to be made freely accessible if your manuscript is accepted for publication. This policy applies to all data except where public deposition would breach compliance with the protocol approved by your research ethics board. If you are unable to adhere to our open data policy, please kindly revise your statement to explain your reasoning and we will seek the editor's input on an exemption. Please be assured that, once you have provided your new statement, the assessment of your exemption will not hold up the peer review process.

*Answer: Thank you for highlighting this important requirement. We fully support open science principles and the sharing of de-identified data. However, due to the highly limited recruitment pool and the sensitive nature of the interview material, the dataset contains potentially identifiable information that cannot be fully anonymized without compromising participant confidentiality. As such, public deposition would not be in compliance with the ethical approval granted for this study.*

*Nevertheless, we are committed to transparency and will make relevant excerpts from the transcripts available upon reasonable request, pending approval from the University of Gothenburg. Researchers interested in accessing such material may contact Professor Karin Ahlberg (Head of Department), who serves as the institutional representative for data access matters, at karin.ahlberg@gu.se.*

*We have updated our data availability statement accordingly and respectfully request an exemption from public deposition in line with PLOS data sharing policy.*

6. Your ethics statement should only appear in the Methods section of your manuscript. If your ethics statement is written in any section besides the Methods, please move it to the Methods section and delete it from any other section. Please ensure that your ethics statement is included in your manuscript, as the ethics statement entered into the online submission form will not be published alongside your manuscript.

*Answer: Thank you. The ethics section has been moved to the Methods section.*

7. Please include captions for your Supporting Information files at the end of your manuscript, and update any in-text citations to match accordingly. Please see our Supporting Information guidelines for more information: http://journals.plos.org/plosone/s/supporting-information.

*Answer: Thank you. We have included captions for Supporting Information files at the end of the manuscript, and updated any in-text citations to match accordingly*

8. [Please remove all personal information, ensure that the data shared are in accordance with participant consent, and re-upload a fully anonymized data set.Note: spreadsheet columns with personal information must be removed and not hidden as all hidden columns will appear in the published file.

Additional guidance on preparing raw data for publication can be found in our Data Policy (https://journals.plos.org/plosone/s/data-availability#loc-human-research-participant-data-and-other-sensitive-data) and in the following article:](http://track.editorialmanager.com/CL0/http:%2F%2Fwww.bmj.com%2Fcontent%2F340%2Fbmj.c181.long./1/010f019806765347-60962b0b-5aea-4311-8f7c-71c0c6cb4f00-000000/Q1c-qkB9fkUqOY6ZJegVCtNNupEwyPXx2srkTQ85XEE=219) <http://www.bmj.com/content/340/bmj.c181.long>[.](http://track.editorialmanager.com/CL0/http:%2F%2Fwww.bmj.com%2Fcontent%2F340%2Fbmj.c181.long./1/010f019806765347-60962b0b-5aea-4311-8f7c-71c0c6cb4f00-000000/Q1c-qkB9fkUqOY6ZJegVCtNNupEwyPXx2srkTQ85XEE=219)

*Answer:* *Due to the highly limited recruitment pool and the sensitive nature of the interview material, the dataset contains potentially identifiable information that cannot be fully anonymized without compromising participant confidentiality. As such, public deposition would not be in compliance with the ethical approval granted for this study.*

9. If the reviewer comments include a recommendation to cite specific previously published works, please review and evaluate these publications to determine whether they are relevant and should be cited. There is no requirement to cite these works unless the editor has indicated otherwise. 
*Answer: Thank you for this clarification.*

Reviewers' comments:

Reviewer's Responses to Questions

**Comments to the Author**

1. Is the manuscript technically sound, and do the data support the conclusions?

The manuscript must describe a technically sound piece of scientific research with data that supports the conclusions. Experiments must have been conducted rigorously, with appropriate controls, replication, and sample sizes. The conclusions must be drawn appropriately based on the data presented.

Reviewer #1: Partly

Reviewer #2: Partly

2. Has the statistical analysis been performed appropriately and rigorously?

Reviewer #1: N/A

Reviewer #2: Yes

3. Have the authors made all data underlying the findings in their manuscript fully available?

The [PLOS Data policy](http://track.editorialmanager.com/CL0/http:%2F%2Fwww.plosone.org%2Fstatic%2Fpolicies.action%23sharing/1/010f019806765347-60962b0b-5aea-4311-8f7c-71c0c6cb4f00-000000/oGdslunTyplubKFNjvVazFQQaVyH4YBIyPoGbK9paAY=219) requires authors to make all data underlying the findings described in their manuscript fully available without restriction, with rare exception (please refer to the Data Availability Statement in the manuscript PDF file). The data should be provided as part of the manuscript or its supporting information, or deposited to a public repository. For example, in addition to summary statistics, the data points behind means, medians and variance measures should be available. If there are restrictions on publicly sharing data—e.g. participant privacy or use of data from a third party—those must be specified.

Reviewer #1: No

Reviewer #2: Yes

4. Is the manuscript presented in an intelligible fashion and written in standard English?

PLOS ONE does not copyedit accepted manuscripts, so the language in submitted articles must be clear, correct, and unambiguous. Any typographical or grammatical errors should be corrected at revision, so please note any specific errors here.

Reviewer #1: Yes

Reviewer #2: Yes

5. Review Comments to the Author

Please use the space provided to explain your answers to the questions above. You may also include additional comments for the author, including concerns about dual publication, research ethics, or publication ethics. (Please upload your review as an attachment if it exceeds 20,000 characters)

**Reviewer #1:**

Thank you for the opportunity to review this interesting article assessing mangers’ experiences of decision making and crisis leadership during the COVID-19 pandemic. The article is well written and easy to follow. However, I have a few questions mainly concerning the methods and use of CFIR framework.

*Answer: Thank you for this positive feedback.*

Major issues
1. Reading the article it is unclear why you choose to use the CFIR framework. To my understanding CFIR is primarily used to guide planned and structured implementation of evidence-based methods. Although it can be used for evaluation the reasoning behind choosing CFIR should be clarified.

*Answer: We thank the reviewer for this valuable comment and agree that our rationale for choosing CFIR needs to be clarified. While CFIR has often been applied in planned and structured implementation efforts, it is also widely used in evaluation and can guide research across the entire process. In their systematic review, Kirk et al. (2016) highlight the value of integrating the CFIR throughout the research process – in study design, data collection, and analysis. This flexibility made CFIR particularly suitable for our study context.*

*During the pandemic, clinical teams could not plan their crisis management in a structured way. By applying a theoretically grounded framework such as CFIR in our study design, data collection, and analysis, we were able to capture critical dimensions of the implementation that the clinicians themselves might not have articulated. We believe that a purely inductive approach would likely not have generated the same depth and nuance. Thus, the use of CFIR allowed us to systematically explore barriers and facilitators under highly unplanned and rapidly changing circumstances, making it a valuable choice for this study.*

*To clarify, the following addition has been made to the methods section on page 8, line 162-167:*

*“ The choice of CFIR was based on its proven flexibility across study design, data collection, and analysis, as highlighted in a systematic review by Kirk et al. (23). During the pandemic, crisis management within maternity care could not be planned in a structured way. Using a theoretically grounded framework made it possible to capture important dimensions of the implementation process that might not otherwise have been articulated, thereby adding depth and nuance that a purely inductive analysis likely would not have achieved.”*

2. The manuscript would gain from more clarity regarding what is seen as the innovation or implementation object in the present study. To me it is unclear if the innovation is the crisis management or several different innovations.

*Answer: Thank you for this clarifying* *question. We have now added the following formulations to the Methods section on line 163 on page 8 to distinguish more clearly between what constitutes the innovation itself and what pertains to its implementation*

*“In our analysis we have defined the crisis management amongst the managers as the innovation, consisting of various measures such as decision-making, implementation of changes, and ways of managing one's own well-being”*

3. In the aim you state that you are exploring “how managerial decisions were made and implemented”. Please define what you mean with implemented in this case as opposed to initiated or adopted.

*Answer: Thank you for this important question. To clarify our interpretation of implementation, we have added the following formulations to the Methods section on line 155-158, page 7.*

*“Regarding the implementation process we consider both initiated and adopted decisions. Initiated is used interchangeably to initiated, commenced/started and launched, although they are not strictly synonymous. And implemented likewise interchangeably with adopted, approved or introduced.”*

4. In the methods section you mention that you used purposeful sampling. Please elaborate on the sampling strategy and what kind of purposeful sampling you used (typical cases, maximum variation etc.).

*Response: We thank the reviewer for this important comment. In the revised manuscript, we have clarified our sampling strategy on page 7 line 129-139:*

*“We applied a purposeful sampling strategy with the aim of achieving maximum variation. Managers were strategically selected to represent different organizational levels (first-line, middle, and senior management), both university and non-university hospitals, and geographical spread across the country. We also sought to include clinics that had made different operational choices during the pandemic (e.g., whether to allow partners on postnatal wards or not) and managers with varying lengths of leadership experience. In some cases, multiple managers from the same organization were included in order to capture variation within local decision-making structures. The sampling strategy was informed by the concept of “information power” (20,21) emphasizing that the richness and relevance of the data, rather than sample size alone, determine the adequacy of the material for the study aim.”*

5. Related to point no. 2 the analysis process needs some more explanation. For example, what was the reasoning behind the results under the heading “innovation domain”? As this domain in CFIR focuses on the qualities of the innovation (it’s evidence base, relative advantage etc.) it is hard to follow when several different innovations are mentioned.

*Answer: Thank you for this clarifying question. We have now added the following formulations to the Methods section on line 159-161 on page 8:*

*“In our analysis we have defined the crisis management amongst the managers as the innovation, consisting of various measures such as decision-making, implementation of changes, and ways of managing one's own well-being.”*

6. The methodological discussion is very brief. I would like to see some discussions regarding preconception and trustworthiness etc.

*Response:* *We thank the reviewer for this important comment. In the revised manuscript, we have expanded the methodological discussion to more explicitly address issues of researcher preconceptions and strategies to ensure trustworthiness, in line with SRQR standards. We describe how the research team’s professional backgrounds provided contextual sensitivity but also required reflexivity during analysis. Furthermore, we clarify how credibility, dependability, confirmability, and transferability were considered, drawing on Elo et al. and adapting their recommendations for a deductive content analysis approach. Specifically, we highlight the use of multiple coders and consensus discussions to enhance credibility, transparent documentation of procedures to ensure dependability, multidisciplinary discussions to support confirmability, and a detailed description of context and sampling to enable transferability. These additions strengthen the methodological transparency of the study.*

*The following paragraph has been added to the discussion, page 18, line 430-446.*

*“The research team’s professional backgrounds inevitably shaped the research process. Several authors had extensive clinical and leadership experience within maternity care during the pandemic, which provided contextual sensitivity but also required reflexivity throughout the analysis. To strengthen trustworthiness, we applied strategies described by Elo et al. (25), focusing on credibility, dependability, confirmability, and transferability, adapted to a deductive content analysis approach. Credibility was enhanced by involving several researchers in the coding process, discussing and reaching consensus on how the CFIR framework was applied, and by illustrating the analytical process with examples. Dependability was supported by a transparent description of the data collection and analytic procedures, enabling other researchers to follow the logic of the study. Confirmability was strengthened by regular discussions within the research team, where different professional perspectives were used to challenge interpretations and ensure that findings were grounded in the data rather than in individual preconceptions. Transferability was addressed by providing a detailed description of the study context and sampling strategy, and thereby allowing readers to judge the relevance for other settings. These strategies, in line with the SRQR standards (29), increase the trustworthiness of the findings and support the validity of the conclusions drawn.”

Minor issues
1. Please clarify if the pilot interview was included in the study or not.*

*Answer: Thank you. The pilot interview was not included in the study, which has been clarified on page 7, line 149 in the methods section:*

*“The test interview was not included in the analysis.”*

*2. The conclusion is not very concise. Some of that is stated in the conclusion can be moved to the discussion.*

*Answer: Thank you for this important comment. We have revised the discussion and conclusion sections and added a “Lessons for Future Crisis Leadership Recommendations” section and a clearer conclusion.*

**Reviewer #2:**

1) Research Area/Title
• The title is overly lengthy and could be more concise. Phrases like "deductive content analysis from the COPE Staff project" are redundant and detract from clarity.
• The focus on "Swedish maternity care managers" is clear, but the title does not immediately highlight the unique contribution of the study (e.g., crisis leadership strategies).
• Suggested title: “Crisis Leadership Under Uncertainty: Decision-Making Strategies of Swedish Maternity Care Managers During COVID-19”

*Answer: Thank you for this constructive feedback and helpful comment. The study is part of a larger research project comprising multiple components published across different journals. We have chosen to include the study name in the titles of all related publications, in line with the practice of several major longitudinal scientific studies. According to the SRQR recommendations we have also stated the methodology of the study in the title. With these two aspects in mind, we have nevertheless aimed to shorten the title and updated the title to better reflect the scope and focus of the study.*

*The revised title now reads:*

*“Crisis Leadership and Strategic Decisions in Swedish Maternity Care During the COVID-19 Pandemic: A Deductive Analysis from the COPE Staff Project”*

2) Research Problems/Gap

• The gap is vaguely stated. While the abstract mentions "limited information on managers’ situation," the introduction does not explicitly critique existing literature or quantify the gap (e.g., lack of studies on maternity care managers in Sweden during COVID-19).

*Answer: Thank you for this helpful suggestion. We have revised the introduction accordingly.*

• The justification for focusing on Sweden (e.g., no lockdown) is buried in the introduction and should be foregrounded as a key gap.

*Answer: Thank you for this helpful suggestion. We have revised the introduction accordingly.*

3) Research Objective/Research Question
 The objective ("generate knowledge about managers’ regulatory strategies") is broad. A specific research question (e.g., "How did Swedish maternity care managers navigate decision-making under pandemic uncertainty?") is missing.

*Answer: Thank you for the feedback regarding the need to narrow the research question for greater clarity. We have reformulated the aim:*

*“Thus, the aim of the study was to generate knowledge on how Swedish maternity care managers navigated decision-making under pandemic uncertainty.”*

• The link between the CFIR framework and the research objective is unclear. Why CFIR? How does it address the gap?

*Answer: We thank the reviewer for this valuable comment and agree that our rationale for choosing CFIR needs to be clarified. While CFIR has often been applied in planned and structured implementation efforts, it is also widely used in evaluation and can guide research across the entire process. In their systematic review, Kirk et al. (2016) highlight the value of integrating the CFIR throughout the research process – in study design, data collection, and analysis. This flexibility made CFIR particularly suitable for our study context.*

*During the pandemic, clinical teams could not plan their crisis management in a structured way. By applying a theoretically grounded framework such as CFIR in our study design, data collection, and analysis, we were able to capture critical dimensions of the implementation that the clinicians themselves might not have articulated. We believe that a purely inductive approach would likely not have generated the same depth and nuance. Thus, the use of CFIR allowed us to systematically explore barriers and facilitators under highly unplanned and rapidly changing circumstances, making it a valuable choice for this study.*

*To clarify, the following addition has been made to the methods section on page 8, line 162-167:*

*“ The choice of CFIR was based on its proven flexibility across study design, data collection, and analysis, as highlighted in a systematic review by Kirk et al. (23). During the pandemic, crisis management within maternity care could not be planned in a structured way. Using a theoretically grounded framework made it possible to capture important dimensions of the implementation process that might not otherwise have been articulated, thereby adding depth and nuance that a purely inductive analysis likely would not have achieved.”*

4) Methodology
• The sampling strategy is not adequately justified. The rationale for selecting 18 managers from 10 hospitals is unclear, with no explanation of how data saturation was determined or whether the sample reflects diversity across roles, institutions, or regions.

*Answer: We thank the reviewer for this important comment. In the revised manuscript, we have clarified our sampling strategy. We used a purposeful sampling approach with maximum variation, aiming to capture managers at different organizational levels (first-line, middle, senior), from both university and non-university hospitals, with geographical spread across Sweden. We also included clinics that had made different operational decisions during the pandemic (e.g., regarding partner presence on postnatal wards) and managers with varying lengths of leadership experience. In some cases, multiple managers from the same organization were interviewed to capture variation within local structures. Furthermore, our approach was guided by the concept of “information power”* (1)*(Malterud et al., Lancet 2016), highlighting that the adequacy of the sample was determined by the richness and relevance of the data for the study aim.*

*In the revised manuscript, we have clarified our sampling strategy on page 7 line 129-139:*

*“We applied a purposeful sampling strategy with the aim of achieving maximum variation. Managers were strategically selected to represent different organizational levels (first-line, middle, and senior management), both university and non-university hospitals, and geographical spread across the country. We also sought to include clinics that had made different operational choices during the pandemic (e.g., whether to allow partners on postnatal wards or not) and managers with varying lengths of leadership experience. In some cases, multiple managers from the same organization were included in order to capture variation within local decision-making structures. The sampling strategy was informed by the concept of “information power” (20,21) emphasizing that the richness and relevance of the data, rather than sample size alone, determine the adequacy of the material for the study aim.”*

• While the paper notes that the CFIR interview guide was modified, it does not provide details on how the questions were adapted or tailored to the study context. This limits transparency and makes it difficult to assess the appropriateness of the interview protocol.

*Answer: Thank you for this important comment. For full transparency, we have provided the interview guide as a supplement to this manuscript.*

• The paper states that 33 out of 48 CFIR constructs were identified in the data, but it does not explain why the remaining constructs were excluded or how their absence might influence the comprehensiveness or interpretation of the findings.

*Answer: Thank you, we understand the need of clarification. It is common that all CFIR constructs do not appear in data when using CFIR as a framework as seen in the review by Kirk et al 2015. To make this clearer, we have added this to the methods section (along with a more thorough description on the data analysis, see comment 6 below).*

*The following has been added to the methods section:*

*“The framework can be applied in a flexible way, and all constructs will not necessarily be present in the data (18)”*

• Although the researchers’ clinical backgrounds—as obstetricians and midwives—may enrich their contextual understanding, this also introduces potential bias. However, issues of reflexivity are not addressed, and the manuscript lacks a discussion of how the researchers’ positionality may have shaped data collection or analysis.

*Response: We thank the reviewer for this valuable observation. In the revised manuscript, we have added a discussion on reflexivity and positionality. We acknowledge that the research team’s professional backgrounds provided in-depth contextual understanding but also carried a risk of bias. To address this, we incorporated strategies to enhance reflexivity and trustworthiness, such as involving several researchers with different professional perspectives in the coding process, engaging in regular discussions to challenge interpretations, and having a trained research assistant with no prior clinical experience conduct the interviews. These measures helped us to critically reflect on how our positionality might have influenced both data collection and analysis. The expanded methodological discussion now explicitly addresses these issues.*

*The following paragraph has been added to the discussion (page 18, line 430-446)*

*“The research team’s professional backgrounds inevitably shaped the research process. Several authors had extensive clinical and leadership experience within maternity care during the pandemic, which provided contextual sensitivity but also required reflexivity throughout the analysis. To strengthen trustworthiness, we applied strategies described by Elo et al. (25), focusing on credibility, dependability, confirmability, and transferability, adapted to a deductive content analysis approach. Credibility was enhanced by involving several researchers in the coding process, discussing and reaching consensus on how the CFIR framework was applied, and by illustrating the analytical process with examples. Dependability was supported by a transparent description of the data collection and analytic procedures, enabling other researchers to follow the logic of the study. Confirmability was strengthened by regular discussions within the research team, where different professional perspectives were used to challenge interpretations and ensure that findings were grounded in the data rather than in individual preconceptions. Transferability was addressed by providing a detailed description of the study context and sampling strategy, and thereby allowing readers to judge the relevance for other settings. These strategies, in line with the SRQR standards (29), increase the trustworthiness of the findings and support the validity of the conclusions drawn.”*

5) Contribution to Knowledge/Practice/Methodology
 • The practical contribution (e.g., "transparency in decision-making") is repetitive and lacks novelty. How do these findings differ from prior crisis leadership studies?

*Answer: Thank you for this important comment. We have revised the discussion section and added a new “Lessons for Future Crisis Leadership Recommendations” section to the discussion on page 18-19 to decrease the repetitiveness, increase the novelty and relate our findings to prior leadership studies.*

• The methodological contribution of using CFIR for crisis leadership is underdeveloped. The discussion should explicitly state how CFIR advanced understanding beyond generic qualitative analysis.

*Response: We thank the reviewer for this important comment. In the revised manuscript, we have expanded the discussion to more clearly articulate the methodological contribution of using CFIR (page 18-19, line 447-463):*

*“A methodological contribution of this study is the application of CFIR in the context of crisis leadership(18,30). While CFIR is commonly used to guide planned implementation processes, it has also been highlighted as valuable for structuring data collection and analysis in diverse study designs(23). In our study, applying CFIR as a deductive framework enabled a systematic exploration of factors influencing managerial decision-making at multiple levels of the healthcare system, spanning innovation, inner and outer setting, individuals, and implementation processes. This provided a structured lens that allowed us to identify not only evident but also less articulated barriers and facilitators that managers themselves may not have expressed without a guiding framework.*

*By anchoring the analysis in CFIR(18), we were able to move beyond a generic thematic description of managers’ experiences and instead situate our findings in relation to well-defined constructs of implementation science. This methodological approach strengthens the transferability of our results, as it links context-specific observations of maternity care managers to a broader conceptual framework. In doing so, the study contributes to methodological development by demonstrating how CFIR can be applied in unplanned crisis situations, thereby offering insights into how implementation frameworks can advance understanding of leadership and decision-making under extraordinary conditions.”*

6) Data Analysis
• The deductive content analysis process is superficially described. Key steps (e.g., coding disagreements, inter-coder reliability) are omitted.
• Table 2 provides an example of coding but does not demonstrate rigor (e.g., frequency of codes, how themes were consolidated).
• The claim of "consensus" among coders is vague. Were disagreements documented? How were they resolved?

*Response: We thank the reviewer for these constructive comments. In the revised manuscript, we have expanded the description of the deductive content analysis process to more clearly demonstrate rigor. We now describe how all interviews were coded by at least two researchers, how a subset was jointly coded to establish a shared approach, and how disagreements were documented and resolved through iterative team discussions. Where necessary, the coding framework was refined to ensure consistency across the dataset. While inter-coder reliability statistics were not calculated—consistent with our qualitative tradition—we strengthened trustworthiness by using systematic consensus-building and multidisciplinary reflexive discussions.*

*In addition, we clarify that Table 3 illustrates the stepwise process from meaning unit to code, construct, and domain. Consolidation of codes into higher-order categories was based on patterns across interviews rather than frequency counts, as our aim was to capture the depth and variation of managers’ perspectives rather than quantify responses. These revisions increase transparency and strengthen the methodological rigor of the study. The methodology section has now been expanded and reads (page 8, line 168-182):*

*“A deductive content analysis, inspired by Elo & Kyngäs (24), was performed informed by the CFIR (18). The analysis followed the recommended phases of preparation, organisation, and reporting. All transcripts were read multiple times by the research team to achieve immersion in the data. A coding framework based on CFIR domains and constructs was developed and refined iteratively. Each interview was coded manually by at least two researchers (ES, SG, AW, KL). A subset of interviews was first coded jointly to establish a common understanding of the framework and to pilot the coding procedure. Discrepancies in coding were documented and resolved through discussion in iterative team meetings until agreement was reached. When consensus could not be reached immediately, transcripts and codes were revisited, and the coding framework was adjusted accordingly. This process ensured that interpretations were not determined by a single researcher’s perspective. Rigour was enhanced through systematic consensus-building and continuous reflexive discussions within the multidisciplinary team. This iterative and collaborative approach strengthened the credibility and dependability of the findings, in line with recommendations for ensuring trustworthiness in deductive content analysis (25).*

*Meaning units were coded into the most relevant CFIR domain and then grouped under constructs. Codes were further consolidated into higher-order categories to capture patterns across managers’ accounts. Table 2 illustrates an example of the coding process, from meaning unit to code, construct, and domain, and reflects the systematic steps undertaken to ensure transparency. Rather than emphasizing frequency counts, which are not central to the study aim, the analysis focused on the richness and variation of the data, in line with qualitative research principles.”*

7) Research Findings and Discussion
• The findings section relies too heavily on direct quotes without sufficient synthesis. For example, the repeated statement that "peer support was essential" lacks deeper analysis, such as the types of support described or how these experiences varied by managerial level.

*Answer: Thank you. We have added a more thorough description of the types of peer support. (page 14 line 326-329*

*“The managers constantly needed to reflect on and evaluate their decisions. Peer support was perceived as a valuable tool. This occurred through both formal and informal management groups, as well as through digital contact with various authorities, specialist associations, and colleagues at departments of obstetrics and gynaecology in different parts of the country. “*

*Furthermore, the findings have been more thoroughly synthesized and discussed in the revised discussion section (see below).*

• The discussion section offers only a limited comparison to existing literature, such as Björk et al. (2022). A more thorough discussion should explicitly contrast the study’s findings with previous research on healthcare leadership during the COVID-19 pandemic.

*Answer: Thank you for this important comment. The discussion has been revised by including a new “Lessons for Future Crisis Leadership Recommendations” on page 18-19 in which the findings are discussed in relation to previous research.*

• The limitations section notes the risk of self-report bias and the lack of employee perspectives, but it does not critically assess how these limitations may have impacted the findings. For instance, the reliance on managerial accounts could have led to an overestimation of leadership effectiveness.

*Answer: We thank the reviewer for this important comment. In the revised manuscript, we have expanded the methodological discussion to more explicitly address issues of researcher preconceptions and strategies to ensure trustworthiness, in line with SRQR standards. We describe how the research team’s professional backgrounds provided contextual sensitivity but also required reflexivity during analysis. Furthermore, we clarify how credibility, dependability, confirmability, and transferability were considered, drawing on Elo et al. and adapting their recommendations for a deductive content analysis approach. Specifically, we highlight the use of multiple coders and consensus discussions to enhance credibility, transparent documentation of procedures to ensure dependability, multidisciplinary discussions to support confirmability, and a detailed description of context and sampling to enable transferability. These additions strengthen the methodological transparency of the study*

*The following paragraph has been added to the discussion (page 18, line 430-446)*

*“The research team’s professional backgrounds inevitably shaped the research process. Several authors had extensive clinical and leadership experience within maternity care during the pandemic, which provided contextual sensitivity but also required reflexivity throughout the analysis. To strengthen trustworthiness, we applied strategies described by Elo et al. (25), focusing on credibility, dependability, confirmability, and transferability, adapted to a deductive content analysis approach. Credibility was enhanced by involving several researchers in the coding process, discussing and reaching consensus on how the CFIR framework was applied, and by illustrating the analytical process with examples. Dependability was supported by a transparent description of the data collection and analytic procedures, enabling other researchers to follow the logic of the study. Confirmability was strengthened by regular discussions within the research team, where different professional perspectives were used to challenge interpretations and ensure that findings were grounded in the data rather than in individual preconceptions. Transferability was addressed by providing a detailed description of the study context and sampling strategy, and thereby allowing readers to judge the relevance for other settings. These strategies, in line with the SRQR standards (29), increase the trustworthiness of the findings and support the validity of the conclusions drawn.”*

8) Conclusion and Implication
• The current conclusion largely restates the results without offering clear, actionable recommendations. Implications should be specific and practical—for example: “Organizations should formalize peer-support networks to support crisis leadership.”
• The suggestion to "incorporate strategies into crisis management frameworks" is too vague. What specific frameworks are being referred to—hospital-level protocols, national emergency guidelines? And how should these strategies be incorporated—through policy updates, training modules, or structural reforms?
• Overall, the implications section is underdeveloped. It does not convincingly explain why the findings matter in a broader context, particularly beyond the COVID-19 pandemic. Given the high volume of pandemic-related research, the paper must clearly answer: Why does this study still matter now?
• While the study identifies important elements like ad-hoc decision-making, peer support, and transparent communication, it falls short of synthesizing these insights into a generalizable, transferable model for managing future healthcare crises—whether pandemics, natural disasters, or staffing shortages.

*Answer: Thank you for your feedback and comments regarding the need for clarification and further specification. We have revised the adding a new section in the manuscript “Lessons for Future Crisis Leadership Recommendations” on page 19-20, which develops the implication section further, discuss our findings against prior research and offering clear, actionable recommendations*

*The added section now states:*

*“****Lessons for Future Crisis Leadership Recommendations***

*Leveraging lessons from the pandemic to identify organizational interventions is crucial for enhancing resilience during future crises and improving managers' working conditions (27,31). Our findings suggest several actionable lessons for future preparedness.*

*First, leaders benefit from rapid yet revisable decision-making protocols that make explicit how decisions are updated as evidence shifts, alongside clear internal communication channels to cascade changes efficiently. This aligns with empirical work underscoring crisis communication competencies in health systems and the need for structured internal communication routines during COVID-19*(2–4)*. Hospital leadership training should incorporate competencies in organizational restructuring to foster agility and responsiveness amid rapid change, using scenario-based modules informed by COVID-19 experiences. This includes revising decision-making structures, clarifying roles and communication channels, and identifying key individuals and competencies.*

*Second, establishing and maintaining peer-support infrastructures (both formal and informal) is critical. In our study, managers actively sought collegial consultation to stress-test decisions and share emerging practices. Evidence from reviews of support interventions for healthcare workers during pandemics indicates that structured emotional/peer support can mitigate distress and enhance perceived capacity to cope. Organizations should formalize peer-support networks to support crisis leadership.*

*Third, developing clear internal communication channels accessible both from home and at work. Digital platforms (for coordination, information dissemination and remote meetings) should be embedded in routine operations to be readily scalable in crises. Reviews and scoping syntheses during and after the pandemic describe how ICT-enabled solutions and telehealth supported remote coordination, monitoring and communication, capabilities that map directly onto the challenges described by managers in this study* (5)*. However, security issues need to be considered even during pandemics when quick solutions are needed* (6)*.*

*Fourth, to address enduring post-pandemic pressures, notably burnout and staffing instability, organizations should pair rapid decision protocols with supports that protect leaders’ well-being (e.g., access to peer networks, transparent governance, and realistic workloads). Some recent multi-year and regional analyses show burnout remains elevated relative to pre-pandemic baselines*(7)*, and systematic reviews report high prevalence across settings*(5)*, underscoring the need for sustained organizational measures rather than episodic crisis fixes .*

*Finally, resilience is strengthened when these practices are maintained in non-crisis periods. Our results highlight leaders’ reliance on transparent communication, rapid feedback loops, and trusted colleagues; recent work on crisis leadership competencies similarly emphasizes anticipatory planning, prioritization, and swift decision capacity* (2)*, competencies that can be cultivated before emergencies occur.*

*Together, these lessons translate our CFIR-informed analysis into practical recommendations: codify dynamic decision-updating and internal communication routines; institutionalize peer-support mechanisms; invest in interoperable digital tools and training; and proactively resource leadership well-being to buffer ongoing workforce strain. These steps are actionable in everyday operations and scalable in future crises.*

*Key recommendations:*

*I.* ***Formalise rapid and revisable decision-making protocols****, including clear communication routines and defined roles.
II.* ***Institutionalise peer-support systems*** *(both formal and informal) to strengthen crisis leadership capacity.
III.* ***Embed interoperable digital communication platforms*** *into routine operations, ensuring secure, accessible coordination in crises.
IV.* ***Safeguard managers’ well-being*** *by combining crisis decision supports with sustained measures against burnout (e.g., realistic workloads, staffing stability, transparent governance).*

9) Recommendations for Improvement
 • A dedicated subsection titled “Lessons for Future Crisis Leadership” should be added to distill the study’s findings into actionable and enduring principles for future preparedness.
• This subsection could highlight key recommendations such as implementing rapid decision-making protocols, establishing peer-support infrastructure, and developing clear internal communication channels.
• The study’s findings should be explicitly connected to ongoing healthcare challenges in the post-pandemic era, including burnout, staffing shortages, and digital transformation.
• The manuscript could explore how maternity care leaders might maintain peer networks and transparent leadership practices during non-crisis periods to enhance resilience.
• Although digital tools are mentioned in the study, their role in long-term preparedness is not fully developed. Expanding on how digital platforms can support remote coordination, triage, or staff communication would improve the study’s practical relevance.
• The discussion should be linked to global frameworks, such as the WHO Health Emergency Preparedness Framework, to emphasize the broader policy significance of the findings.
• The manuscript should also clarify the specific policy or practice gaps it addresses. The current conclusion refers vaguely to “incorporating strategies into crisis management frameworks” without specifying whose frameworks are being considered or how integration should occur.
• Providing concrete examples—such as recommending that hospital leadership training programs include scenario-based modules based on COVID-19 lessons—would significantly enhance the clarity and applicability of the study’s implications.

*Answer: Thank you for these excellent ideas. We are very grateful for these concrete and valuable suggestions, and we have revised the manuscript accordingly, see above.*

*____________________________________________________*
