# Supplementary material for: Crisis leadership and strategic decisions in Swedish maternity care during the COVID-19 pandemic: A deductive analysis from the COPE staff project
Source: PLoS One. 2026 May 22;21(5):e0346625. doi: 10.1371/journal.pone.0346625 (PMC13196918; doi:10.1371/journal.pone.0346625)
Supplement: Attachment — Submitted filename: Response to reviewers_revision 2_Leading in crisis.docx [file pone.0346625.s007.docx]

**Response to reviewers**

*Answer: Thank you for the opportunity to revise this manuscript. The authors would like to thank the editor and the reviewers for the time they have devoted to this process and for their important and constructive comments, which we believe have significantly contributed to improving the quality of the manuscript.*

*Please find our point-to-point responses below.*

**Reviewer #1:**

I believe the revised version shows improvement in several areas. However, I still have some questions, particularly regarding the choice of framework and its application. You present interesting material and results that are worth disseminating, but I would encourage you to reconsider the use of an implementation framework in this study.

*Answer: Thank you for this positive feedback and for recognizing the value of the material and results. We appreciate the opportunity to further clarify our reasoning regarding the choice and application of the framework. We agree that this is not a typical study for the use of CFIR, and we acknowledge that this choice therefore warrants careful justification in light of your concerns.*

*First, we consider the use of a theoretical framework to be important in studies of this kind, as it can help capture analytical dimensions that may not be explicitly articulated by participants themselves. While inductive approaches are valuable, we judged that a purely inductive analysis might have limited our ability to systematically explore factors influencing decision-making under highly complex and rapidly changing conditions. When reviewing the emerging literature from the pandemic, we also noted that many studies were primarily observational and descriptive, with limited analytical structuring. In this context, the use of a framework offered a way to move beyond description and support analytical interpretation with potential relevance for policy and practice. We fully recognize that CFIR is not the only possible framework for such an analysis. At the time of study initiation, however, it was the most relevant framework available to us, and the urgency of securing data collection during the pandemic in order to capture real-time experiences and decision-making meant delaying data collection to explore alternative frameworks risked affecting data quality, for example through recall bias or changing organizational conditions. Our findings suggest that CFIR may have utility beyond its traditional areas of application in this specific context, although we agree that such use should be interpreted with caution.*

*Second, implementation processes differ substantially across fields. In clinical contexts, standardized and well-defined interventions, along with implementation processes aimed at improving patient health or clinical treatment, often represent the gold standard. In contrast, interventions aimed at improving employees’ occupational health do not follow the same logic. Instead, the importance of adapting both intervention measures and the implementation process itself has been emphasized, see for example von Thiele Schwarz, U., Nielsen, K., Edwards, K., Hasson, H., Ipsen, C., Savage, C., … Reed, J. E. (2021). How to design, implement and evaluate organizational interventions for maximum impact: the Sigtuna Principles. European Journal of Work and Organizational Psychology, 30(3), 415–427.* [*https://doi.org/10.1080/1359432X.2020.1803960*](https://doi.org/10.1080/1359432X.2020.1803960)*. From this perspective, the crisis management processes examined in our study can be understood as an organizational-level work environment intervention shaped by local conditions. Adaptation is therefore not a methodological weakness but a key principle in organizational workplace interventions. Applying an implementation framework such as CFIR, which has demonstrated its value in generating in-depth knowledge in evaluation studies, allowed us to systematically examine these adaptive and context-dependent processes.*

*Finally, the interview guide and analytical strategy were developed on the basis of CFIR from the outset. Replacing the framework post hoc and re-interpreting the data through a fundamentally different analytical structure could introduce methodological uncertainties and biases and reduce coherence between data collection and analysis, with potential implications for the study’s trustworthiness. For these reasons, we have chosen to retain the current analytical approach. In response to your comments, we have clarified our rationale and strengthened our acknowledgement of limitations in the manuscript, and we hope that these revisions address your concerns.*

Below I have pasted my original comment, your answer to them and then my new comment.

Major issues

1. Reading the article it is unclear why you choose to use the CFIR framework. To my understanding CFIR is primarily used to guide planned and structured implementation of evidence-based methods. Although it can be used for evaluation the reasoning behind choosing CFIR should be clarified.

Answer: We thank the reviewer for this valuable comment and agree that our rationale for choosing CFIR needs to be clarified. While CFIR has often been applied in planned and structured implementation efforts, it is also widely used in evaluation and can guide research across the entire process. In their systematic review, Kirk et al. (2016) highlight the value of integrating the CFIR throughout the research process – in study design, data collection, and analysis. This flexibility made CFIR particularly suitable for our study context. During the pandemic, clinical teams could not plan their crisis management in a structured way. By applying a theoretically grounded framework such as CFIR in our study design, data collection, and analysis, we were able to capture critical dimensions of the implementation that the clinicians themselves might not have articulated. We believe that a purely inductive approach would likely not have generated the same depth and nuance. Thus, the use of CFIR allowed us to systematically explore barriers and facilitators under highly unplanned and rapidly changing circumstances, making it a valuable choice for this study.

To clarify, the following addition has been made to the methods section on page 8, line 162-167:

“ The choice of CFIR was based on its proven flexibility across study design, data collection, and analysis, as highlighted in a systematic review by Kirk et al. (23). During the pandemic, crisis management within maternity care could not be planned in a structured way. Using a theoretically grounded framework made it possible to capture important dimensions of the implementation process that might not otherwise have been articulated, thereby adding depth and nuance that a purely inductive analysis likely would not have achieved.”

New comment: I agree that the use of CFIR should be integrated throughout the research process. However, as Kirk et al also points out it should be used in implementation studies with identified implementation outcomes. In this study the implementation object consists of various measures and the outcome is not specified. I think using an implementation framework in a study with the aim “to generate knowledge on how Swedish maternity care managers navigated decision-making under pandemic uncertainty” is questionable as the aim is not to evaluate the implementation of an intervention.

*Answer: Thank you for these clarifications to the original comment. As noted above, implementation processes differ substantially between clinical contexts—where the focus is on patient care—and organizational-level workplace interventions, which focus on employees’ occupational health. In general, managers are responsible for conducting context‑specific occupational health and safety management to prevent ill‑health among employees, in accordance with Swedish work environment legislation. This routine work is supported by internal functions such as HR departments, which possess substantial expertise in implementing work environment interventions. These resources also supported the crisis management efforts during the pandemic instructing them to perform a context‑adapted crisis management process, including designing and implementing context‑specific preventive measures to maintain operability and ensure a sustainable working environment for employees, in accordance with recommendations for organizational‑level workplace interventions.*

*To make this clearer, we have added the following statement to the Methods section:*

*“**Instead, the managers were* *instructed to perform a context‑adapted crisis management process, including designing and implementing context‑specific preventive measures to maintain operability and ensure a sustainable working environment for employees, in accordance with recommendations for organizational‑level workplace interventions.”*

*In addition, after reviewing the study aim, we agree with the reviewer that it could be improved. We have revised the aim to more clearly articulate the purpose and scope of the study. The new aim reads:*

*“Thus, the aim of the study was to generate knowledge on how Swedish maternity care managers navigated decision‑making under pandemic uncertainty by evaluating the implementation of crisis management.”*

*Lastly, we fully agree that this* *is not a typical study for using CFIR and should therefore be interpreted with caution. To clarify, an addition has also been made to the limitation section:*

*“It should also be noted that the CFIR framework has most commonly been used to guide planned and structured implementation of evidence-based methods, rather than context-adjusted organisational work-environment interventions. In this study, CFIR 2.0 was applied in a context-sensitive and partial way to structure managers’ accounts of crisis management. The findings should therefore be interpreted with this specific application and context in mind.”*

2. The manuscript would gain from more clarity regarding what is seen as the innovation or implementation object in the present study. To me it is unclear if the innovation is the crisis management or several different innovations.

Answer: Thank you for this clarifying question. We have now added the following formulations to the Methods section on line 163 on page 8 to distinguish more clearly between what constitutes the innovation itself and what pertains to its implementation “In our analysis we have defined the crisis management amongst the managers as the innovation, consisting of various measures such as decision-making, implementation of changes, and ways of managing one's own well-being”

New comment: As for point no. 1 – do you really evaluate the implementation of a specific intervention or are you more interested in how crisis management was conducted and perceived during the pandemic (which is important and interesting, although not for implementation science)?

*Answer: Thank you for this thoughtful clarification and for engaging further with the alignment between the study aim and the use of an implementation framework. We appreciate your point regarding the importance of clearly defined implementation objects and outcomes when applying CFIR, as emphasized by Kirk et al., and we agree that this is an important consideration.*

*In this study, the implementation object does not consist of a single, standardized intervention but rather of a set of context-adapted crisis management measures developed and enacted by managers in response to pandemic-related uncertainty. As such, the implementation outcomes are not framed as predefined endpoints but are reflected in how these measures were initiated, adapted, and sustained in practice under rapidly changing conditions. Our intention was therefore not to evaluate the effectiveness of a specific intervention, but to examine the processes through which crisis management was implemented and navigated in an organizational context. We recognize that this positions the study somewhat different from more conventional implementation evaluations, and we have sought to clarify this distinction more explicitly in the manuscript.*

*In response to your comment, we have revised the study aim to better reflect this focus and to more clearly articulate that the study examines decision-making and crisis management through the lens of implementation processes, rather than evaluating a discrete intervention with predefined outcomes. We have also expanded the methodological description and limitations section to acknowledge that CFIR is most commonly applied in studies of planned and structured implementations, and that its use in the present context requires cautious interpretation.*

*We hope that these clarifications help to address your concerns regarding the fit between the study aim, the analytical framework, and the type of implementation processes examined, and we are grateful for your suggestions, which have helped us refine both the framing and transparency of the study.*

3. In the aim you state that you are exploring “how managerial decisions were made and implemented”. Please define what you mean with implemented in this case as opposed to initiated or adopted.

Answer: Thank you for this important question. To clarify our interpretation of implementation, we have added the following formulations to the Methods section on line 155-158, page 7.

“Regarding the implementation process we consider both initiated and adopted decisions. Initiated is used interchangeably to initiated, commenced/started and launched, although they are not strictly synonymous. And implemented likewise interchangeably with adopted, approved or introduced.”

New comment: What I was aiming for with my question was whether crisis leadership was actually implemented, or rather spread on its own (diffusion), and was used in different ways by different managers?

*Answer: Thank you for this clarification, which helps us better understand the intent behind your question. We agree that an important distinction can be made between crisis leadership being formally implemented, spreading through diffusion, and being enacted in different ways by different managers.*

*In the context of this study, crisis leadership was not introduced as a standardized or uniform model to be rolled out identically across settings. Rather, managers were tasked with carrying out a context-adapted crisis management process, which included designing, initiating, and enacting preventive and organizational measures suited to their local conditions in order to maintain operability and support a sustainable working environment for employees. This meant that crisis leadership practices were indeed applied and operationalized, but in ways that varied across managers and settings.*

*We therefore understand the processes examined in this study as implementation of crisis management at the organizational level, while fully acknowledging that this implementation involved substantial local adaptation and variation in how leadership practices were enacted in practice. We have clarified this distinction in the Methods section to better reflect that implementation, in this case, refers to the enactment and adaptation of crisis management measures rather than the uniform adoption of a predefined leadership model.*

*We hope this clarification addresses your question regarding the distinction between implementation, diffusion, and variation in leadership practices.*

5. Related to point no. 2 the analysis process needs some more explanation. For example, what was the reasoning behind the results under the heading “innovation domain”? As this domain in CFIR focuses on the qualities of the innovation (it’s evidence base, relative advantage etc.) it is hard to follow when several different innovations are mentioned.

Answer: Thank you for this clarifying question. We have now added the following formulations to the

Methods section on line 159-161 on page 8: “In our analysis we have defined the crisis management amongst the managers as the innovation, consisting of various measures such as decision-making, implementation of changes, and ways of managing one's own well-being.”

New comment: I think the use of CFIR makes it hard to follow the results. How do you for example evaluate the evidence strength and quality, relative advantage, adaptability and complexity of crisis leadership in general? The results under “innovation domain” are more a description of how the managers tackled the situation than reflecting for example the evidence strength of the innovation (crisis leadership). Same for the other domains.

*Answer: Thank you for this careful and important comment. We appreciate your observation that, in this study, the use of CFIR may make the results more challenging to follow, particularly in relation to how constructs within the innovation domain are commonly understood.*

*We agree that constructs such as evidence strength and quality, relative advantage, adaptability, and complexity are often applied to clearly specified and evidence-based innovations. In line with CFIR 2.0, however, innovations may also consist of complex and evolving sets of practices rather than single, standardized interventions. In the present study, the innovation was defined as crisis management as an organizational response, comprising multiple, context-adapted measures enacted by managers under conditions of uncertainty and limited available evidence.*

*Accordingly, our analysis within the innovation domain did not aim to evaluate the formal evidence base or effectiveness of crisis leadership as a predefined intervention. Rather, consistent with a CFIR 2.0 perspective, the framework was used as an analytic structure to organize and interpret how managers described, reasoned about, and enacted crisis management practices in real-world settings. The results presented under the innovation domain therefore reflect managers’ perceptions and experiences of these practices, rather than an assessment of evidence strength or comparative advantage in a traditional evaluative sense.*

*To support transparency and readability, we have clarified this analytical approach in the Methods section, where we now explicitly state that CFIR 2.0 was used as an analytic framework to structure the analysis, rather than to evaluate the effectiveness or evidence base of a predefined intervention. We have also strengthened the discussion of limitations to acknowledge that this represents a context-sensitive and partial application of the framework. We hope that these clarifications address your concerns regarding the interpretation of the innovation domain and the presentation of results across CFIR domains.*

**Reviewer #2:**

The authors have made substantial and thoughtful improvements to the manuscript, directly addressing nearly all the critical points raised in the previous review. The revisions have significantly strengthened the paper's clarity, methodological rigor, theoretical contribution, and practical relevance. The manuscript is now more focused, better justified, and positioned to make a meaningful contribution to the literature on crisis leadership in healthcare.

*Answer: Thank you for this positive and extensive feedback on the revision and resulting manuscript, which we truly appreciate.*

1. Research Area/Title

The title has been significantly improved to: "Crisis Leadership and Strategic Decisions in Swedish Maternity Care During the COVID-19 Pandemic: A Deductive Analysis from the COPE Staff Project". While the new title is more direct and includes the key concepts ("Crisis Leadership," "Strategic Decisions"), it remains somewhat long. However, it accurately reflects the study's content and is acceptable. The core issue from the previous comment (redundancy, lack of focus) has been largely resolved. The suggested shorter title could still be an option, but the current one is adequate.

*Answer: Thank you for this positive feedback. We agree that the current title is somewhat long. At the same time, we aimed to balance conciseness with clarity, in line with the journal’s author guidelines, which emphasize that titles should be specific, descriptive, and comprehensible to readers outside the field, and include the method used. While we explored alternative shorter formulations, we found that further shortening risked reducing precision or omitting key elements of the study. We therefore chose to retain the current title.*

2. Research Problems/Gap

The introduction now explicitly and clearly states the research gap (lines 84-99): It highlights the lack of exploration of leadership in "persistent crises" in maternity care, the scarcity of studies on Swedish healthcare managers during COVID-19, and the unique Swedish context (no lockdown). The gap is now well-defined, contextualized, and compelling. It clearly justifies why this study is necessary and novel.

*Answer: Thank you for this positive feedback.*

3. Research Objective/Research Question

The aim is now precisely stated: "to generate knowledge on how Swedish maternity care managers navigated decision-making under pandemic uncertainty" (lines 106-107). The rationale for using CFIR is explicitly justified in the methods section (lines 100-105, 162-167). The authors explain that CFIR helps identify factors influencing implementation outcomes and, despite being designed for planned implementation, its flexibility allows it to capture important dimensions of the unplanned crisis management process. The research objective is now specific and focused. The justification for using CFIR is clear and strengthens the methodological foundation.

*Answer: Thank you for this positive feedback.*

4. Methodology

The purposeful sampling strategy is now clearly described with a strong rationale (lines 128-139), citing "information power" and detailing the criteria for maximum variation (organizational level, hospital type, geography, operational choices, experience). Interview Guide is noted as a "modified version of the CFIR interview guide," with a test interview conducted. The Supporting Information includes the guide, enhancing transparency. The paper acknowledges that "all constructs will not necessarily be present in the data" (line 153), which manages expectations. The finding that 33 of 48 constructs were identified is presented as a result, not a flaw. Researcher Reflexivity & Bias is now robustly addressed (lines 120-127, 430-446). The authors detail their clinical backgrounds, state how this was considered during analysis, and explain that interviews were conducted by a research assistant without healthcare ties to mitigate bias. The trustworthiness section (lines 430-446) thoroughly discusses credibility, dependability, confirmability, and transferability. The methodology section is now transparent, rigorous, and defensible. The handling of reflexivity is particularly commendable.

*Answer: Thank you for this positive feedback.*

5. Contribution to Knowledge/Practice/Methodology

A new "Methodological contribution" subsection has been added to the discussion (lines 447-463). It explicitly argues for the value of applying CFIR to crisis leadership, allowing a systematic, multi-level analysis that revealed both evident and less-articulated factors. The practical contributions (transparency, peer support, communication) are now more effectively synthesized and linked to the CFIR domains. The methodological contribution is now a key strength of the paper. The study convincingly demonstrates how CFIR can be applied beyond its traditional use.

*Answer: Thank you for this positive feedback. We appreciate this assessment and have aimed to present the application of CFIR transparently, including a clear discussion of its scope and limitations in this context.*

6. Data Analysis

The data analysis section (lines 168-189) now provides a much more detailed, step-by-step account of the deductive content analysis process. It describes preparation, coding by multiple researchers, consensus-building through iterative meetings, handling discrepancies, and the consolidation of codes. It emphasizes rigor through systematic consensus-building and reflexive discussions within the multidisciplinary team. The description now meets standards for qualitative rigor, demonstrating how the analysis was conducted and how trustworthiness was ensured.

*Answer: Thank you for this positive feedback.*

7. Research Findings and Discussion

The results are now structured logically by CFIR domain, with effective synthesis and interpretation alongside the quotes. The narrative moves beyond simple description to explain patterns and variations (e.g., different approaches to protecting pregnant staff). The discussion is substantially expanded and deepened. It now:

Engages more thoroughly with relevant literature (e.g., Björk et al., 2022; Kniffin et al., 2021) to compare and contextualize findings.

Introduces and applies the Demands-Resources model (lines 360-379) as a theoretical lens to analyze managers' experiences, which is a significant analytical upgrade.

Critically examines the implications of managers' strategies, such as the organizational vulnerability created by relying on personal time (lines 410-415).

The discussion is now analytical, theory-informed, and critically engaged with the literature. It successfully interprets the findings within a broader scholarly conversation.

*Answer: Thank you for this positive feedback.*

8. Conclusion and Implication & 9. Recommendations for Improvement

The authors have added a comprehensive new section: "Lessons for Future Crisis Leadership Recommendations" (lines 464-516). This section is exemplary and directly addresses the core weakness of the previous version. It provides five specific, actionable lessons (rapid/revisable decision protocols, peer-support infrastructures, digital communication channels, protecting leader well-being, maintaining practices in non-crisis periods), each supported by references to the study's findings and external literature. These are distilled into four clear "Key Recommendations" (lines 507-516) for formalizing protocols, institutionalizing support, embedding digital tools, and safeguarding well-being. The conclusion (lines 517-521) is now concise and effectively points to the need to incorporate these strategies into frameworks. This is the most impactful revision. The paper transforms from a descriptive case study into a forward-looking source of evidence-based guidance for policy and practice. It answers the "so what?" question convincingly.

*Answer: Thank you for this positive feedback.*
